# Adonis Vernalis

**Published:** 1892-07-30

**Authors:** 


### THERAPEUTICS.

### ADONIS VERNALIS.

Adonis vernalis has been before the profession since the early part of the sixteenth century, and during the last ten or twelve years its properties and uses have been made the subject of investigation by many writers of distinction.

From the U.S. Dispensations we note that it belongs to the family of Ranunculaceæ, and is found in the North of Europe and Asia, where it has been used as an abortifaciant. Linderos examined the leaves and found in them ten per cent. of aconitic acid. Dr. Cervello, in 1882, obtained from this plant a glucoside, to which he gave the name of adonidin. This glucoside occurs in the form of a somewhat hygroscopic, canary-yellow powder of an intensely bitter taste; soluble in water, alcohol, and amylic alcohol. According to Dr. Cervello and Dr. H. A. Hare, adonidin at first slows the action of the frog's heart, increasing at the same time the force of the systole, and finally produces arrest. Dr. Hare states that this arrest is diastolic; Dr. Cervello, that it is systolic. Both experimenters found that in mammals adonidin increases very markedly the arterial pressure, whilst decreasing the pulse rate, and that after toxic doses the primary rise is followed by a marked fall of arterial pressure, with irregularity of the heart's action. The primary rise of presure appears to be chiefly cardiac, although there is some reason for supposing that the drug does exert some influence upon the vaso-motor system. The first slowing of the pulse was found by Dr. Hare to be due to stimulation of the inhibitory nerves, as it was prevented by their previous section, whilst finally the fall of pressure was at least in great part due to the vaso-motor palsy. In 1879 the Adonis vernalis was introduced to the medical world as a cardiac stimulant by Bubnow. Since then it has been tested by a number of physicians with fairly concordant results. The general testimony is, that its action in disease resembles that of digitalis, and that it is useful in the same class of cases. It is much more prompt than digitalis, and it is affirmed by Durand to have no cumulative tendency. There have been some differences of opinion in regard to its diuretic action. Whatever such influence, it must be attributed to the effect upon the circulation in the kidneys rather than to any marked direct power over the secreting structure.

Marfori and Borgiotti believe that adonis is most serviceable in cases of heart disease accompanied with marked venous engorgement, when it is desirable to regulate the work and produce prolonged diuresis. They employed it mostly in cases of mitral regurgitation and mitral stenosis. Its action has been investigated by Dr. Oliver* in cases of mitral and aortic regurgitation, and in all the patients relief was afforded to the precordial pain, to palpitation, and dyspnœa.

In seven cases in which adonidin was given there was relief to all the unpleasant symptoms. Every patient who has taken it expresses himself, after a few days' trial, as feeling generally very much better. The painful throbbing of blood vessels, headache, and profuse perspirations have, in addition to dyspnœa and precordial pain, disappeared; and although there have been in most of the cases a slight increase in the amount of urine, Dr. Oliver thinks that adonidin has but little diuretic action. He is of opinion that it is a cardiac tonic acting chiefly upon the heart, gently raising arterial tension, thus agreeing with the results of the physiological investigations to which we have already alluded. But it further seems to have some sedative action upon the heart, and the cases of aortic regurgitation in which it seems to answer best are those where the lesion is due either to traumatic rupture of the valve, or to chronic aortitis, and where it has not arisen from rheumatic endocarditis.

As regards the dose of adonidin, Durand put the dose at about one-third of a grain, every three or four hours. Dr. Oliver gave one-sixth of a grain dose of adonidin four times daily in chloroform water. Bubnow employed the infusion made from the whole herb, four to eight parts in 180 parts of water; of this the dose was a tablespoonful every two hours. Borgiolti prescribed it in the form of an infusion, of which the dose was one to two drachms.

It will be seen, then, that in adonis we appear to have an efficient cardiac stimulant resembling digitalis, but having a less marked action on the peripheral arterioles, and standing midway between digitalis and strophantum.

The remedy has been employed by Dr. A. N. Lavrovski†, whose observations were made on ten male patients suffering from dropsy, who were kept in a ward together in the Alexandra Hospital in St. Petersburg. The special object of this research was to discover the effects of adonis on the insensible loss from the lungs and skin in dropsical cases. For this purpose the observations were divided into three periods of several days each, during each of which the ingesta and egesta were carefully weighed and examined; together of course with weighing the patients, the insensible loss could be accurately ascertained. It was found that not only did the drug, which was given in the form of infusion, act as a heart tonic and as a diuretic, but that it also increased the insensible loss, which, however, did not equal the urine excreted. The action in both respects was somewhat slow in establishing itself, and when the drug was left off things very soon returned, more or less approximately, to their previous condition. The effect, however, was more prolonged in cases where the dropsy was of cardiac than where it was of renal origin. As a rule it was found that the insensible loss was greater day by day than by night. One or two examples may be quoted. A man with weak heart had, before taking adonis, a daily insensible loss of 840 grammes; during the twelve days he took the drug his average loss was 885 grammes; and during the subsequent eight days 831 grammes. The mean daily quantities of urine passed in the three periods were 3,056, 3,247, and 2,645 grammes respectively. In another and more serious case, diagnosticated an insufficiency of the aortic valves, with arteriosclerosis and dilatation of the ascending aorta, the effects, both in the insensible loss and in the urine, were more marked, the average of the former being 802, 1,127, and 892 grammes during the three periods respectively, and of the latter, 1,363, 2,202, and 1,814 grammes.

* *Lancet*, November 28th, 1888. † *Lancet*, August 29

